# The Current Status of Enteric Fever Diagnostics and Implications for Disease Control

**DOI:** 10.1093/cid/ciaa503

**Published:** 2020-07-29

**Authors:** Stephen Baker, Christoph J Blohmke, Mailis Maes, Peter I Johnston, Thomas C Darton

**Affiliations:** 1 Cambridge Institute of Therapeutic Immunology and Infectious Disease, Department of Medicine, University of Cambridge, Cambridge, United Kingdom; 2 Data Science and Clinical Systems, GSK Vaccines, Siena, Italy; 3 Florey Institute for Host-Pathogen Interactions, Department for Infection, Immunity and Cardiovascular Disease, Faculty of Medicine, Dentistry and Health, University of Sheffield, Sheffield, United Kingdom

**Keywords:** enteric fever, typhoid, diagnostics, *Salmonella*, global health

## Abstract

Enteric (typhoid) fever remains a problem in low- and middle-income countries that lack the infrastructure to maintain sanitation and where inadequate diagnostic methods have restricted our ability to identify and control the disease more effectively. As we move into a period of potential disease elimination through the introduction of typhoid conjugate vaccine (TCV), we again need to reconsider the role of typhoid diagnostics in how they can aid in facilitating disease control. Recent technological advances, including serology, transcriptomics, and metabolomics, have provided new insights into how we can detect signatures of invasive *Salmonella* organisms interacting with the host during infection. Many of these new techniques exhibit potential that could be further explored with the aim of creating a new enteric fever diagnostic to work in conjunction with TCV. We need a sustained effort within the enteric fever field to accelerate, validate, and ultimately introduce 1 (or more) of these methods to facilitate the disease control initiative. The window of opportunity is still open, but we need to recognize the need for communication with other research areas and commercial organizations to assist in the progression of these diagnostic approaches. The elimination of enteric fever is now becoming a real possibility, but new diagnostics need to be part of the equation and factored into future calculations for disease control.

Enteric (typhoid) fever is a clinical syndrome caused by ingestion of the gram-negative bacteria *Salmonella enterica* serovar Typhi (*S*. Typhi) or *Salmonella enterica* serovar Paratyphi (*S*. Paratyphi) A, B, or C. Despite the availability of good antimicrobial treatment regimens and new-generation conjugate vaccines for preventing *S.* Typhi infection (TCVs), enteric fever continues to cause a significant degree of morbidity and mortality worldwide; current estimates suggest approximately 13.5–26.9 million new cases of *S.* Typhi and *S.* Paratyphi A (the focus of this review) each year [1, 2]. The uncertainty around these estimates is, in part, due to the limitations in availability and performance of current diagnostic tools.

Enteric fever is associated with a broad spectrum of clinical disease, ranging from asymptomatic to severe symptoms, such as fever, malaise, headache, and complications of ileal ulceration including perforation and profuse hemorrhage. Furthermore, some individuals who are exposed to *S*. Typhi or *S*. Paratyphi A will go on to become long-term carriers, where the organisms are retained in the gallbladder and occasionally at other sites including the kidney. Why different individuals present with differing clinical symptoms is not understood, but laboratory models and human challenge with virulent organisms have revealed a highly complex natural history of infection resulting from the co-evolution of bacteria with humans. The result is that both *S*. Typhi and *S*. Paratyphi A are exquisitely adapted to transit almost effortlessly throughout the human body, triggering a cascade of events that result in symptomatic disease in some, and long-term asymptomatic carriage in others. Consequently, diagnosing enteric fever remains an enigma, with symptomatic infections mimicking various aspects of many other infectious diseases. These factors are especially relevant in settings with the limited resources found in low- and middle-income countries (LMICs), where differential diagnostic tests (many of which have their own inherent limitations) for febrile disease are often not available, and diagnosis is performed purely on the clinical judgment of an attending healthcare worker [3].

Inaccurate diagnosis of enteric fever (and other common febrile diseases) results in the under- or overdiagnosing of infectious pathologies and frequently unsuitable treatment. The knock-on effects of inaccurate diagnosis include inappropriate antimicrobial prescribing and suboptimal clinical management, which may contribute to the development and preservation of antimicrobial resistance. Additionally, a lack of a reliable diagnostic results in public health policymakers being supplied with poor-quality data, posing challenges to the effective evaluation or introduction of new interventions, including potentially effective vaccination strategies [3–5].

Almost all techniques for diagnosing an infectious disease focus on (1) the direct detection of the pathogen of interest (either the entire pathogen through culture or a fragment of it through a molecular process), or (2), the indirect detection of the pathogen via a measurement derived from the host response to the infecting agent, indicating recent exposure or active infection. The development of new rapid diagnostics for enteric fever has been a challenge for decades, with blood culture remaining the only real widely used standard against which new tests and intervention strategies are evaluated. The performance of blood culture in detecting *S.* Typhi/*S.* Paratyphi A varies markedly and requires automated culture systems and an adequate volume of blood to be most effective [4]. Even in highly controlled experimental settings, the sensitivity of automated blood culture may only reach 80% [6]. A recent meta-analysis measured a diagnostic sensitivity of 59% (95% confidence interval, 54%–64%), when compared to the seldom used, true gold standard of bone marrow aspirate culture [7]. Various efforts have been made to improve the accuracy of the standard against which to perform diagnostic evaluations, including the use of composite endpoints or Bayesian latent class modeling analysis [8–10].

In parallel to new research methods, there has been a resurgence of interest in exploring new approaches to identifying the cause of fever in patients in LMICs. The ready availability of access to blood and/or serum in patients may facilitate the application of novel high-throughput methods. These methods remain largely agnostic, aiming to identify disease-specific signatures or biomarkers indicating recent or active infection, and are principally conducted as a component of a comprehensive research program, not least due to the bulk of information acquired with the attendant requirement for computing and bioinformatic processing ability. The overall aim of such programs is to exploit such datasets to obtain highly detailed and individualized data not available through previous studies, and is only possible now due to access to, and decreasing costs of, many high-throughput molecular technologies. Parallel advances are required in biological annotation, mathematical modeling, and computational analysis techniques that will lead to an unprecedented array of approaches in which to interrogate the large and complex datasets that are generated. Here, we describe several such new and innovative approaches for the identification and early validation of acute enteric fever through transcriptomics, metabolite profiling, and the humoral response to the organisms. We discuss these new techniques and their pros and cons as we move into a new era of typhoid control and elimination.

## SEROLOGICAL RESPONSES

The detection of an antibody response indicating recent infection or exposure using an easily accessible and standardized biological sample substrate (such as serum or plasma) remains an attractive approach for enteric fever diagnostics. The hypothetical advantages to serological methods include avoiding the infrastructure (including reagents, equipment, and laboratory capacity) and training required to perform culture-based diagnostics as well as the potential rapidity of such methods. Realistically, at the time of writing, if we are to have a new universal point-of-care diagnostic test for enteric fever within the next 5 years, it will likely be based on a serological assay via a lateral flow platform. The ease of use of lateral flow systems, the ability to combine the reagents for an enteric fever assay with tests for other fever-causing pathogens, and the amount of data that can be obtained from a small volume of easily accessed biological sample currently make serology the most likely short-term solution. Technologies exist to move into this area now, but we still lack suitable, reproducible serological targets. While several advances have been made over the poorly performing Widal test, which has now been in use for > 100 years, these have largely been minor incremental gains, resulting in a new generation of serological diagnostic tests that still produce unacceptable levels of specificity and sensitivity for clinical and epidemiological use. Several remaining limitations of these serological diagnostics stem from the narrow repertoire of antigen/antibody combinations (targets) that are unique to *S.* Typhi/*S.* Paratyphi [11] and their downstream suboptimal evaluation and reporting in appropriate populations or clinical studies [12].

The problem faced by researchers in determining which target(s) to use for new serological tests for enteric fever is demonstrated by human challenge study data from nonendemic settings [6]. In the Oxford challenge model, healthy adult volunteers ingest a single dose of the challenge organism (*S.* Typhi Quailes strain) and are closely monitored over 14 days for disease and recovery. The collection of serum samples at multiple time points before and after challenge followed by probing with a panel of approximately 4500 *Salmonella* antigens demonstrated the broad and highly heterogenous antibody response to *S.* Typhi exposure and infection [13]. The response to *Salmonella* infections in endemic populations is potentially amplified by prior exposure occurring throughout childhood, but it remains highly complex and equally difficult to disaggregate.

To navigate the limitations of trying to identify the most indicative antigen/antibody combinations, several groups have used protein microarray platforms to down-select targets in both endemic and nonendemic populations, with subsequent exploration and validation by standard enzyme-linked immunosorbent assay and other approaches designed to detect antigen-specific antibody [14]. The most promising targets identified through these approaches and the populations in whom these have been further explored and validated include immunoglobulin G (IgG) and immunoglobulin A (IgA) responses to hemolysin E and *S.* Typhi–specific lipopolysaccharide (LPS) in pediatric cases in Nigeria [14], immunoglobulin M and/or IgG responses to hemolysin E, the cdtB protein (a component of typhoid toxin) in patients with enteric fever in Bangladesh [15], and IgA responses to *S.* Typhi–specific LPS and cell invasion protein sipC and IgG against hemolysin E in the Oxford human challenge study [13]. The targets identified in these latter studies went on to be validated in patients with natural infection in Nepal [13]. A remaining issue with use of individual antigen targets is the potential for cross-reactivity with other commensal or pathogenic organisms.

The key take-home messages from these studies is that serological tests to diagnose enteric fever are feasible but not as straightforward as predicted and that such approaches are likely to be even more challenging to validate convincingly in endemic patient populations. The major limitations to routine clinical use include the current need for follow-up sampling, unless unequivocal population standard titers for comparison can be demonstrated, and the current inability to infer antimicrobial susceptibility to guide treatment in the era of increasing antimicrobial resistance. Recent studies employing unbiased analysis for candidate antigen/antibody selection have revealed a possible route toward a point-of-care serological test for enteric fever, in particular through the use of combination panels incorporating multiple antigen targets with both polysaccharide and protein antigens, and the ability to detect multiple antibody isotypes. These approaches seem to increase the sensitivity and specificity, decrease the potential effects of the heterogeneity in antibody response, and provide some indication into how recently the infection occurred, thus preventing the requirement for additional samples taken at multiple time points.

## TRANSCRIPTOMICS

Transcriptomics (functional genomics) is the systematic measurement of gene expression at a specific time point in a given cellular compartment. The quantity of each transcript is measured by calculating the amount of messenger RNA transcribed from genomic DNA (ie, gene expression) using any one of a number of high-throughput molecular technologies, such as microarrays and RNAseq. Transcriptomic profiles generally consist of thousands of individual quantitative data points providing a high-resolution, cross-sectional snapshot of the host response to a given stimulus such as infection or vaccination. By annotating genes with biological functions, and incorporating them into common pathways, these profiles can be used as a proxy for interpreting the broad response state of the cell community in that compartment, such as within polymorphonuclear cells in peripheral blood. Patently, cell-specific signatures may be difficult to disaggregate given the mixed populations of cell types that may be represented in the assayed sample. Profiles can be generated from a comparatively small quantity of biological sample (eg, whole blood) [16]. Consequently, transcriptomic science has evolved in recent years and been exploited to generate detailed insights into the human transcriptional response to numerous infectious diseases [17–22] and other alternative immune perturbations [23–26]. These studies produce a fairly standardized output and generate large amounts of data, which are available through public repositories (eg, GEO or ArrayExpress), making them readily accessible for detailed downstream cross-study analysis.

The wealth of available data in transcriptomics studies provides a unique opportunity for identifying diagnostic signatures and, when combined, these datasets are more than sufficient to apply advanced analysis algorithms, such as supervised learning approaches, to identify transcriptional patterns that are specific to any given infection or disease state [27]. Such potential was demonstrated elegantly by Herberg et al [28]; this group generated a large dataset by combining transcriptional profiles in publicly available databases generated from febrile children in Europe and North America. The authors identified a reproducible transcriptional signature able to distinguish viral from bacterial infections in these febrile children. This study performed logistic regression using an elastic net algorithm in combination with a forward-selection partial least square feature selection approach, yielding a 2-gene signature (*FAM89A* and *IFI44L*) able to distinguish bacterial from viral causes of febrile illness with high accuracy [28, 29].

The Oxford human challenge model has been the source of several large gene expression datasets, which were generated in a highly controlled setting, permitting the interrogation of the human transcriptional responses to infection with both *S.* Typhi and *S*. Paratyphi A [21, 22, 30]. More recently, we were able to augment these data with transcriptional data from blood culture–confirmed enteric fever cases in Nepal to generate a unique dataset using the standardized laboratory methods [31]. In this proof-of-concept study, this dataset was amalgamated with transcriptional profiles generated from blood taken from individuals suffering from uncomplicated dengue fever, blood-stage malaria infection with *Plasmodium falciparum*, and active pulmonary tuberculosis. Using a random forest algorithm with integrated feature selection, we identified 5 genes (*STAT1*, *SLAMF8*, *PSME2*, *WARS*, and *ALDH1A1*) that were able to distinguish culture-confirmed enteric fever cases from the other infections with > 96% accuracy in an independent validation cohort. While the infections chosen as comparators may not be universally optimal in enteric fever–endemic settings, these data outline 3 important points. First, this study provides evidence that the host response is a feasible source of biomarkers that are robustly specific to identify enteric fever patients. This observation contrasts the traditional and more intuitive approach to diagnostic biomarker discovery for infections, where the test aims to directly detect the pathogen of interest via culture, polymerase chain reaction (PCR) amplification, or antigen detection. Second, this study further confirms that gene expression profiles are specific enough to potentially distinguish diseases with similar clinical presentation, such as undifferentiated fever syndromes [32]. Third, this approach could lend itself to the detection of multiple different infections. There may be limited commercial value in developing a test to identify an infection caused by 1 pathogen, and the ability to combine multiple targets with suitable specificity realistically appears more reliable and clinically advantageous.

The studies distinguishing bacterial and viral infections, and the enteric fever investigation described here, highlight that a combination of high-resolution human transcriptional response data (generated from human samples using high-throughput technologies) with advanced analytics, including machine learning methods, are powerful tools to identify novel diagnostic biomarkers for infectious diseases and may represent the future of molecular diagnostics for enteric fever.

## METABOLOMICS

Metabolomics is a comparatively new area of scientific research, which has evolved with our ability to detect and measure minute quantities of small chemicals in complex biological material using cutting-edge mass spectrometry technologies [33]. Through the detailed analysis of biological material, metabolomics provides holistic overview of the metabolites (small molecules that are intermediates and downstream products of metabolic pathways) held in the analyzed compartment. In contrast to transcriptomics, which only measures relative transcription, metabolic profiling provides an insight into the direct chemical physiology within the sample and a greater understanding of biological processes in action. In diagnostic application, metabolomics provides chemical signatures that arise through cellular processes subverted through the disease process. Cellular processes act in equilibrium; therefore, when a pathogen interacts with the host and the host produces a cellular response, such as inflammation or initiation of adaptive immune mechanisms, this equilibrium may be impacted. Additionally, infecting organisms activate their own specific cellular pathways to induce infection resulting in the potential modification of downstream metabolites. Metabolomics aims to detect these subtle changes in cellular processes and, unlike serology and transcriptomics, may be able to provide both direct and indirect evidence of the pathogen being present and of its impact on the host.

Metabolomics has been applied to various infectious diseases and sample types, identifying signatures of urinary tract infections in urine, inflammatory disease in cerebrospinal fluid, and viral infections in serum samples [34–36]. Furthermore, the technique may also be able to predict disease outcomes, progression, and even disease onset, as was recently reported for tuberculosis [37]. Given the inherent issues with the diagnosis of enteric fever, metabolomics may offer an alternative approach by providing an exceptionally high-resolution snapshot of a patient presenting with a fever of unknown origin. We were able to exploit this technique to generate metabolite signatures on plasma samples by 2-dimensional gas chromatography with time-of-flight mass spectrometry from 50 patients with enteric fever (25 with *S*. Typhi and 25 with *S*. Paratyphi A) against 25 afebrile controls [38]. An extension orthogonal partial least squares with discriminant analysis model was fitted to differentiate the various groups and 306, 324, and 58 metabolite peaks were found to significantly separate controls from the *S.* Typhi infections, controls from the *S.* Paratyphi A infections, and the *S.* Typhi infections from *S.* Paratyphi A infections, respectively. Notably, the potential of this technique was highlighted by the ability to separate infections caused by the 2 serovars by a collection of specific chemicals, which included phenylalanine, pipecolic acid, and 2-phenyl-2-hydroxybutanoic acid. Furthermore, a metabolite profile comprising 6 classified metabolites (ethanolamine, gluconic acid, monosaccharide, phenylalanine, pipecolic acid, and saccharide) had high discriminatory ability for all enteric fever patients, at least in this set of Nepali patients. This study was later replicated in patient samples from Africa and an additional cohort of patients in Asia [39]. The principal message from this secondary study was that at least 24 metabolites can identify enteric fever (*S*. Typhi only in the secondary study), and included the biologically plausible chemicals, glycerol-3-phosphate (carbon source and precursor for phospholipid biosynthesis), stearic acid (component of liposome), and linoleic acid (bactericidal activity), pyruvic acid, and creatinine. Last, by employing these techniques (sample processing, chemical analysis, and mathematical modeling), we were additionally able to disaggregate *S*. Typhi and *S*. Paratyphi A carriers from the profiles generated from the plasma of the noncarriers [40].

The limited available data suggest that metabolite profiles are a potential future solution for diagnosing enteric fever, but there are many challenges in making these indicative chemical signatures routinely accessible in LMICs. First, there needs to be expansion of available data for enteric fever and validation of current hits in differing laboratories. This primary step is underway as a function of the Strategic Typhoid alliance across Africa and Asia study (STRATAA) study, which is being conducted in Nepal, Bangladesh, and Malawi. As described in the STRATAA protocol, plasma and urine samples from blood culture–confirmed enteric fever patients will be processed to validate the preliminary chemical hits and for additional exploration [41]. Second, the already available hits need to be studied and simple assays need to developed that can detect and quantify these small chemicals in plasma. This secondary step requires the input of chemists and organizations that have the proven experience of developing new technologies for diagnostic tests. Third, metabolite profiling arguably provides the most “revolutionary” approach over other new diagnostic techniques and could easily be scaled up to incorporate other disease etiologies and pathologies. For this approach to work, there needs to be substantial investment in metabolite profiling of multiple different infections and the development of diagnostic chemical panels and a platform on which they can be measured. This standardization seems unlikely at present, but the development of clinically accessible mass spectrometry tools such as matrix-assisted laser desorption/ionization–time of flight technology has revolutionized bacterial identification in clinical microbiology laboratories in less than a decade. The development of a standard approach that can diagnose any infection would be the long-term aspiration of every infectious disease clinician, and metabolomic profiling may be a suitable methodology for which to challenge this aim.

## OTHER DIAGNOSTIC APPROACHES

While there is some interest in the technical challenge of developing better diagnostic tools for enteric fever, the question remains as to whether these can improve over clinical acumen alone. As a syndrome, the symptoms of enteric fever are nonspecific with a wide differential diagnosis. Certain features, such as rose spots, relative bradycardia, and “typhoid tongue,” may be indicative, but their presence is far from universal [42]. Outbreaks of enteric fever in the United States in the 1980s reported varying rates of rose spots (5%–30%) [43]. In Turkey, an attempt was made to develop a prediction model for typhoid from a cohort of patients presenting persistent fever, using culture of *S*. Typhi from stool, blood, bone marrow, or urine plus a consistent clinical presentation as diagnostic, and contrasting with febrile patients who did not meet the case criteria [44]. Age (< 30 years), abdominal distention, confusion, leukopenia, relative bradycardia, a positive Widal test, and typhoid tongue were included in the final prediction model. The presence of 4 or more of these features was associated with a sensitivity of > 85% and specificity of > 78%. Unfortunately, the proportion of participants with this number of features was not reported, but it is improbable that many “real-world” patients would possess these features.

In reality, community and hospital clinics in low-resource tropical settings are inundated with patients presenting with undifferentiated febrile illness every day. The number of these cases peaks in the monsoon or “green” season and, while the majority of patients may be children or younger adults, many may also have underlying medical comorbidities or socioeconomic risk factors such that a wide range of differential clinical diagnoses needs to be taken into account, of which only one will be enteric fever. While parallel surveillance programs may provide support with identifying common prevailing causes of fever and some susceptibility data to guide therapeutic selections, these are expensive to maintain or a result of external influence, including research studies, vaccine introduction surveillance, or enhanced vigilance surrounding an outbreak. In these settings, rapid and affordable, multipathogen point-of-care tests could play a major role in individual patient management and disease control but could also alleviate considerable healthcare strain in coping with this patient load.

Aside from newer technologies, several other laboratory techniques remain the focus of research toward developing better enteric fever diagnostics, including molecular detection of bacterial genes using PCR. PCR techniques are the current mainstay of infectious disease molecular- and non-culture-based diagnostics and, in the case of enteric fever, are frequently designed to detect the flagellin genes of *S*. Typhi and *S*. Paratyphi A, or other more specific targets [45, 46]. The attraction of PCR-based approaches includes the ability to detect bacterial nucleic acid, which does not depend on recovery of living organism and therefore remains theoretically advantageous, especially in settings where antimicrobial use in the community is high. However, any pathogen-directed test for *S.* Typhi/*S.* Paratyphi A remains restricted by the low concentration of bacteria in the peripheral blood and perhaps inherent inhibitors in the biological sample of choice [47]. Techniques to lyse red blood cells have shown some promise in increasing yield [48], and removing human DNA from samples may produce an additional yield, thereby further improving sensitivity and specificity by reducing competing targets [49].

Loop-mediated isothermal amplification (LAMP) is a technique that permits the amplification of nucleic acid at a constant temperature, removing the need for thermal cyclers and a permanent electricity supply. LAMP targeting a specific *S.* Typhi gene produced a positive signal for all positive blood cultures and no (or few, depending on the primers used) false positives in a clinical evaluation [50, 51]. The sensitivity of the technique was < 40% (again, primer dependent) when performed on peripheral blood samples taken from the same patients [51]. Quantitative PCR, using an existing and more standardized approach on the same samples, performed similarly. The technique may give more immediate results than blood culture, but does not improve sensitivity and is severely limited by restricting the assay to detect individual pathogens targets.

While multiple studies have assessed PCR-based approaches, frequent limitations include methodological issues that cannot exclude contamination, study design that fails to include sufficient reference standard samples, or absence of direct testing of clinical material. Studies in which high sensitivities have been reported should remain guarded for these described limitations. In summary, no standardized PCR technique exists, and procedures to optimize sensitivity add expense and complication to an already costly procedure. Such limitations, and the fact that the concentration of nucleic acid in the blood is often below a detectable yield, makes PCR in its current format unsuitable for use in endemic settings where enteric fever tests need to have the greatest sensitivity and specificity.

## OUTLOOK

The field of clinical diagnostics is rapidly evolving and newer technologies, analytical advances, and funder interests are having a major impact in driving their application to the study, management, and control of human and veterinary diseases. Diagnostics for infectious diseases have also moved forward, but arguably not at the same rate as in the areas of oncology, cardiology, and metabolic disease. New bacteriological methods and molecular approaches are slowly being introduced into clinical microbiology laboratories in higher-income countries, but many of these techniques are not directly transferable to LMIC settings with fewer resources. Realistically, outside of major surveillance studies and vaccine implementation programs, we are no better at routinely diagnosing or measuring typhoid incidence than we were 25 years ago. The reasons for this disappointing reality are multifaceted and associated with the biological and technical complexity of the challenge, the lack of a harmonized response (including coordination between different investigators and geographical areas, between investigators, funders, and biotechnological companies, and between different disease control modalities, specifically treatment and prevention measures), poor study design, a focus on other priorities in the enteric fever field (ie, TCV licensure and introduction), and a potential lack of a commercially viable enteric fever–only product after the research and development phase.

The roll-out of TCV in Asia and Africa means that we are entering a new period of enteric fever control. If TCV works as predicted, it is likely to have a major impact on disease burden and transmission of *S.* Typhi, creating a platform for potential elimination. However, we cannot afford to become complacent as many challenges still exist and we are now in a race against time. Multidrug-resistant strains are commonplace in sub-Saharan Africa and Asia and extensively drug-resistant variants have emerged in Pakistan, requiring updated management strategies. There also may be political complications in delivering TCV in all locations, and the vaccine does not protect against *S*. Paratyphi A or other forms of invasive *Salmonella.* The lack of a standardized easy-to-use diagnostic test as an alternative, or in addition, to blood culture, has restricted and will greatly limit our ability to control enteric fever more quickly; which could have major repercussions in containing the effects of antimicrobial resistance and ultimately in reaching the goal of disease elimination.

The typhoid (enteric fever) field has unfortunately missed a prime opportunity to develop and release a diagnostic tool alongside the introduction of new vaccines. A potential consequence of this oversight is the return of the same issues that have hindered vaccine introduction in the past, namely an inability to accurately assess disease burden and measure long-term vaccine effectiveness. We ignore the need for new tools (alternative vaccines, diagnostics, and treatments) for enteric fever at our peril and we reiterate the need for a sustained field-wide push to effectively diagnose, control, and eliminate the disease in endemic areas.

## Notes

***Supplement sponsorship.*** This supplement is funded with support from the Coalition against Typhoid Secretariat, housed at the Sabin Vaccine Institute in Washington, DC and made possible by a grant from the Bill & Melinda Gates Foundation.

***Potential conflicts of interest.*** The authors: No reported conflicts of interest. All authors have submitted the ICMJE Form for Disclosure of Potential Conflicts of Interest.
